# Fluidic Active Transducer for Electricity Generation

**DOI:** 10.1038/srep15695

**Published:** 2015-10-29

**Authors:** YoungJun Yang, Junwoo Park, Soon-Hyung Kwon, Youn Sang Kim

**Affiliations:** 1 Program in Nano Science and Technology, Graduate School of Convergence Science and Technology, Seoul National University, 1 Gwanak-ro, Gwanak-gu, Seoul 151-742, Republic of Korea; 2 Advanced Institutes of Convergence Technology, 864-1 Iui-dong, Yeongtong-gu, Suwon-si, Gyeonggi-do 443-270, Republic of Korea; 3 Flexible Display Research Center, Korea Electronics Technology Institute, 25 Saenari-ro, Bundang-gu, Seongnam-si, Gyeonggi-do 464-816, Republic of Korea

## Abstract

Flows in small size channels have been studied for a long time over multidisciplinary field such as chemistry, biology and medical through the various topics. Recently, the attempts of electricity generation from the small flows as a new area for energy harvesting in microfluidics have been reported. Here, we propose for the first time a new fluidic electricity generator (FEG) by modulating the electric double layer (EDL) with two phase flows of water and air without external power sources. We find that an electric current flowed by the forming/deforming of the EDL with a simple separated phase flow of water and air at the surface of the FEG. Electric signals between two electrodes of the FEG are checked from various water/air passing conditions. Moreover, we verify the possibility of a self-powered air slug sensor by applying the FEG in the detection of an air slug.

Flows in small size channels or tubes can usually be seen in nature, daily life and technology. For example, blood vessels in our body, the phloem and xylem in plants, and ground water in soil spaces or cracks are commonly found flows in nature. Artificially, we also see flows in channels or tubes not only in daily life like water lines in a purifier or heat exchanger but also in technology advancements such as lab on a chip technology, fluidic electronics and water cooling systems in high power electronics. These various flows in small channels and tubes have potential as a source of energy. For flows in small channels as an energy source, many researchers have studied not only the diverse and abundant sources in nature and daily life but also integration capability^1^ and applicability to the fields of biology^2^, medical^2^,^3^,^4^ and chemistry^5^.

Specially, interest in electricity generation with flows in a channel has been recently increased in the fields of micro energy harvesting and sensing^6^,^7^,^8^,^9^,^10^. Several techniques such as variation of capacitance^6^,^7^, reverse electro-wetting^8^ and electro kinetic flow^9^,^10^ have been studied. However, there are several drawbacks to the variation of capacitance and reverse electro-wetting techniques, which are the necessity of external sources such as a bias voltage or pre-charging processes to maintain a voltage difference in capacitor^6^,^7^,^8^. The energy generation of electro kinetic flows uses a pressure gradient in nano-sized channel arrays as a streaming potential^9^,^10^, which needs complicated fabrication processes such as a micro- or nano-patterning process for nano-channel arrays. These challenging points still remain for many applications of electricity generation using flows in a channel.

Recently, we reported on a novel active transducer for effective electricity generation via various water motions without any bias voltage sources^11^,^12^. Based on this concept, herein, we propose a new fluidic electricity generator (FEG) by modulating the electric double layer (EDL) with two phase flows of water and air without any external power source. The driving force of electricity generation in the FEG is assumed that the ion dynamics, which are induced by motion modulations between electrodes and water, make an electricity. To verify the assumption, electric signals between two electrodes of FEG were checked for various water/air passing conditions, such as entering and leaving on channels, and passing into channels with different lengths. Moreover, we verified the possibility of a self-powered air slug sensor by applying the FEG in the detection of an air slug.

## Results

### Fabrication of the FEG and electrical performances.

**Figure 1:**
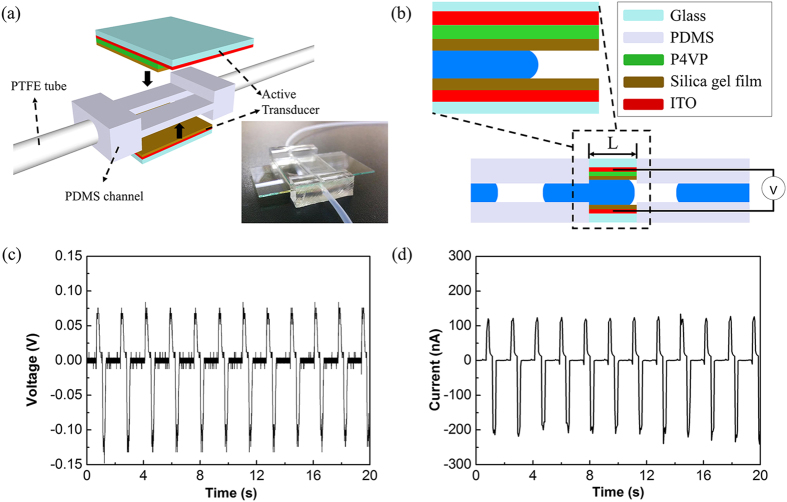
Schematic images and electrical properties of fluidic electricity generator. (**a**) Schematic image and photograph of the FEG. (**b**) Side view of the FEG which includes the PDMS channel, water/air and components of the active transducer. L is the length of the channels (L: 1.8 cm and 3.6 cm). (**c**) The measured output voltage and (**d**) current of the FEG with 10 cm of 0.01 M NaCl water solution and 30 cm air sequence flowing through a 3.6 cm length channel. The two phases of water and air had a flow rate of 30 ml/min with a syringe pump.

Figure 1a shows a schematic image and photograph (inset) of the FEG. A sandwich structure of electrode/PDMS/electrode was adopted for the FEG. Two types of PDMS channels (18 mm and 36 mm long) were fabricated with a 2 mm × 1.9 mm cross-section in both. The top electrode consisted of ITO glass as an electrode, P4VP (poly 4-vinyl phenol) as a dielectric layer and a silica gel layer that included perfluoroalkyl-silane as a hydrophobic layer. Each layer was spin-coated and baked at 200 °C. For the bottom electrode, only silica-gel film that included perfluoroalkyl-silane was coated on the ITO glass by spin coating. The details of the experimental conditions are explained in the Methods. The PDMS channel mounted with two electrodes was closed by a three prong clamp and connected to the PTFE tubes with 1.6 mm inside diameter. An inlet tube was connected to the segmented sequences of the water/air generating system, and an outlet tube was connected to a syringe pump. All the flows were operated in the suction mode of the syringe pump. Both the top and bottom electrodes were connected to measuring equipment as shown in the schematic image of Fig. 1b. Figure 1c,d shows the measured output voltages and currents while the two phase sequences consisted of a 10 cm column of 0.01 M NaCl water solution and 30 cm air slide continuously in the FEG at a flow rate of 30 ml/min. The positive voltage and current signals were measured when the head of a water column moved into the channel, and negative signals were measured when the tail of a water column left the channel. The peak to peak voltage and current were ~0.19 V and ~0.331 μA, respectively.

### Basic mechanism of the fluidic generation system.

**Figure 2:**
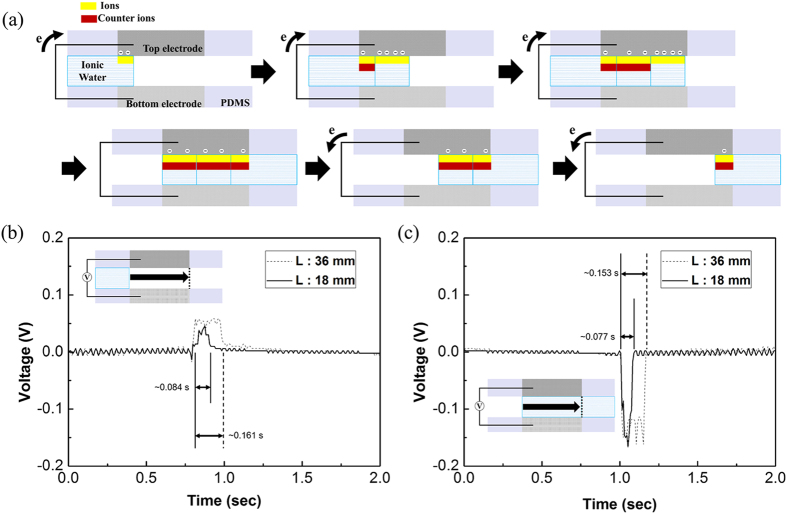
Schematic images of the mechanism and generating time with different length channels. (**a**) Basic mechanism of the FEG. Yellow layer is the attracted ions and red layer is the counter ions. (**b**) Positive voltage signal was measured when the head of a water stream moved into the channels and (**c**) negative voltage was measured when the tail of a water stream left the channels. The measured output voltage for the 18 mm and 36 mm length channels with a water stream flow rate of 30 ml/min are represented by the red and blue lines, respectively.

Figure 2a explains the basic mechanism of electricity generation in the FEG. The driving force of electricity generation in the FEG is assumed to be EDL modulation at the interface between the transducer and water^11^,^12^,^13^. When the 0.01 M NaCl water solution contacts the top and bottom electrodes, cations are adsorbed at the top and bottom electrodes. Electrons and counter ions come near to the electrodes’ surface for charge neutralization. At that time, the cations are asymmetrically adsorbed by the silica-gel coated P4VP film in the top electrode in the FEG^11^,^12^, and the electrons flow through the external circuit, which generates electric currents. In the case of water leaving, the reverse process occurs in which the adsorbed ions are detached by the mechanical motion of the water column. The hold electrons by cations return through the external circuit and generate reverse electric currents. For electricity generation in the FEG, the voltage difference between bottom electrode and ground was almost zero. This result indicates that few electrons were induced by the cations at the bottom electrode, (see Supplementary Fig. S2) and the effects were negligible. Therefore, the voltage difference between the top and bottom electrodes can be expressed as 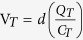 (see the Supplementary Fig. S3), where *C*_*T*_ is the capacitance of the EDL which is formed between the top electrode and water and *V*_*T*_is the voltage on *C*_*T*_. The charge *Q*_*T*_ can be expressed by multiplying *C*_*T*_*and V*_*T*_. In this equation, the variation area of the EDL is an important factor for electricity generation. It means that the flow of the charges is induced by increasing/decreasing the contact area between the water and the silica-gel coated P4VP film on the top electrode in the FEG.

To verify this assumption, the holding time of the voltage difference between the top and bottom electrodes were checked when the water was passing through the channels which have different lengths. According to the hypothesis, the voltage difference should be maintained while the water column passes through the FEG channel. Therefore, the time it takes for the water column to pass through the different lengths of channels should be different. Figure 2b,c shows the measured output voltage versus time. The dash line is the measured voltage data at a flow rate of 30 ml/min for the 3.6 cm long channel when the water went in and out. The solid line represents the voltage data for the 1.8 cm long channel. The voltage maintenance time when the head of the water entered for the long channel case was ~0.161 s and for the short channel case was ~0.084 s shown in Fig. 2b. When the tail of the water column left the channels, the maintenance time was ~0.153 s and ~0.077 s for the long and short channel, respectively. When the water columns passed with same flow rate, more time was required to reach the end of the active transducer in the longer channel shown as the dotted line on the inset images of Fig. 2b,c. These results indicate that the flow of charges was induced by increasing/decreasing the contact area between the water and silica-gel coated P4VP film on the top electrode in the FEG.

The maintenance voltage signals were measured like a noise. The 17.9 and 26.9% of voltage variations measured during the voltage differences maintained when the water column passed through for the 3.6 cm long channel at the flow rate of 30 ml/min. These signals come from remaining tiny water droplets on the electrodes which were formed by the former water columns as a footprint, covered some part of the electrode surface. (see Supplementary Figure S4). For that reason, the irregularity of wetting and dewetting occurred intermittently in the channel, which causes a fluctuation in the signals. For the entering/leaving processes shown Fig. 2b,c, the peak voltage for the leaving case was larger than that for the entering case. For the phenomena, it was assumed that the amount of released electrons by the mechanical motion of the water at the leaving process is larger than the amount of electrons by the adsorbed ions at the entering process. Thus, the peak voltage at the leaving case was higher than that of the other one.

### Electrical properties for energy harvesting.

**Figure 3:**
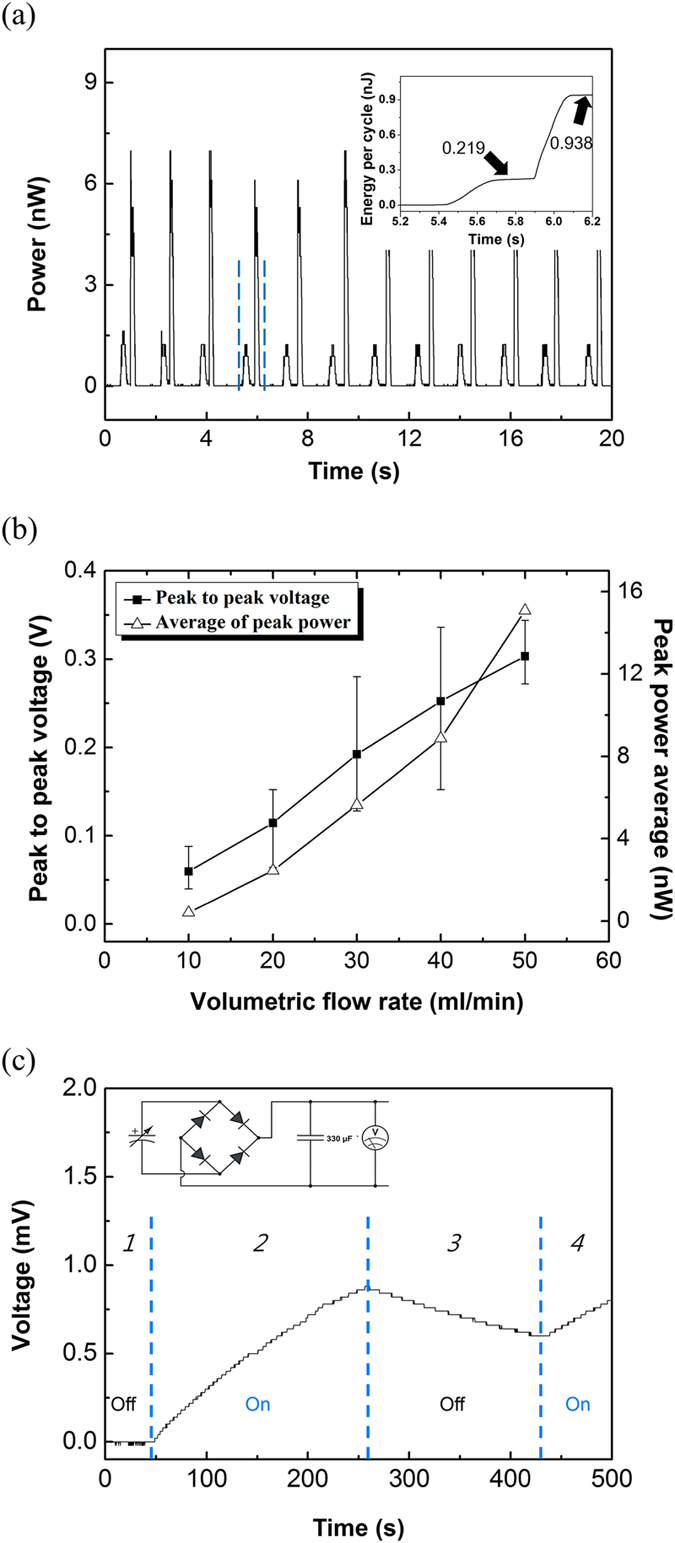
Electric properties of the FEG as an energy harvester. (**a**) The output power when multiple columns of water (10 cm) and air (30 cm) moved with a volumetric flow rate of 30 ml/min. Inset plot shows the generated energy per a cycle. (**b**) The measured peak to peak voltages and peak power averages at different volumetric flow rates. (**c**) The voltage data from a storage circuit (inset) containing a capacitor and a full wave rectifier.

The FEG was tested as an energy harvester with a two phase flow. Figure 3a shows the output power when multiple water columns flowed through the FEG. A 2.2 MΩ external road resistor was used to measure power. There were two peak power signals per cycle shown in the data of Fig. 3a. As the water columns entered the channel case, the measured power was ~1.22 and ~7 nW as the water columns left the channel. The average peak power was ~5.64 nW with 10 cm long water and a 30 cm air sequence with a flow rate of 30 ml/min, ~0.94 nJ per water column was measured (Fig. 3a inset). To characterize efficiency of our FEG device, the calculation was conducted and the energy conversion efficiency of the FEG was confirmed about 1.5% (see Supplementary Note 1). Figure 3b shows that the output voltage and power depended on the velocity of the water columns. The voltage and power increased with an increasing flow rate of the water column. In case of the slow flow rate, the amount of increasing the charge Q_T_ is also small. Therefore, there is enough time to neutralize the charge imbalance near the electrodes. On the other hand, in case of the fast flow rate, the large amount cations adsorbed on the electrodes in a short time before the counter ions come to the electrodes’ surface for the charge neutralization. It means that the larger variation of the contact area per second induces higher power and energy. The inset image of Fig. 3c shows a simple energy storage circuit containing a full wave rectifier and a 330 μF capacitor. The voltage of the capacitor was monitored with a voltmeter while multiple water and air sequences passed through the FEG for 560 s. The same conditions of length of the water and air in the output power experiment were used in the charging experiment. The capacitor was fully discharged before the test (Fig. 3c, zone 1). During the operation of the FEG, the output voltage increased at a rate of 41.7 μV/s (Fig. 3c, zone 2). In the zone 3, leakage loss occurred because of circuit components leakage when the flow stopped operating. Resuming the flow, the water columns contacted the FEG again, and the capacitor was charged as shown in zone 4. From these results, we successfully generated electricity from a two phase flow.

## Discussion

**Figure 4:**
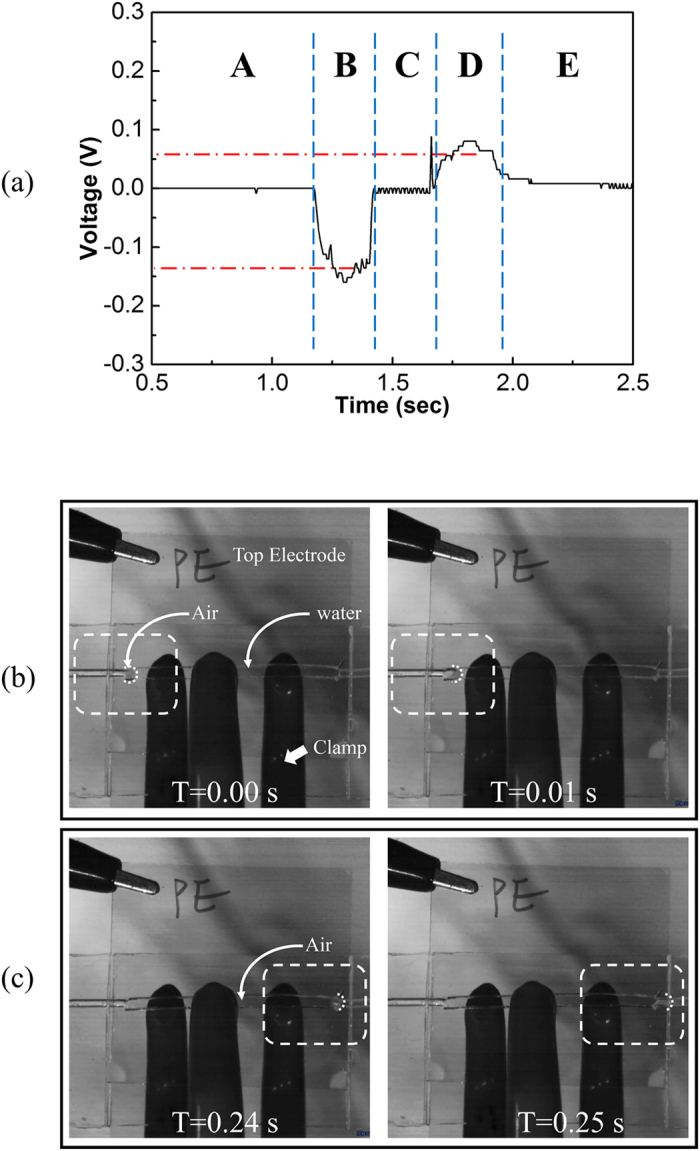
Plot and captured images of the slug flow sensor test (**a**) Application of the FEG for a segmented flow sensor with a two phase flow. (**b**,**c**) High-speed camera images of the flowing water column from top view.

Two phases of water and air flow are widely used in various fields such as the cooling systems of high power electronics, medical devices^14^ and micro reactors^15^ for chemical analysis because the flows have high mass and heat transfer rates in tubes and micro channels^16^,^17^. In these applications, the slug length and speed of the segmented flow have very important roles that affect the mass and heat transfer between phases. A monitoring device for slug length and speed is a necessary part of a two phase flow adopted system, and optical^18^,^19^ and electrical methods^20^,^21^ have been suggested as monitoring systems for segmented flow. With this in mind, we checked the possibility of using the proposed FEG as a self-powered air slug sensor. For the sensing test, a 3.6 cm long FEG, air slug, and 0.01 M NaCl solution were used. To verify the velocity of the slug flow, captured images from a high-speed video camera (MotionPro x4^TM^, Imaging Solutions GmbH) were used. The length of the air gap between the water columns prepared was 12 cm long (0.2616 ml), and the volumetric flow rate was 30 ml/min. Figure 4a shows the output voltage and the divided five zones (A ~ E) according to distinctive features when the air gap and water stream passed through the FEG. At zone A, there was no potential difference between the top and bottom electrodes because the FEG channel was filled with the first water column and just flowed without any variation in contact area. When the head of the air slug (tail of the first water column) entered the FEG channel (Fig. 4b,c), a decrease in the contact area between the water and FEG caused a negative peak shown in Fig. 4a, zone B for several milliseconds. Zone C had no signal because of the passing region of the air slug. When the head of the second water stream (end of the air slug) approached the channel (Fig. 4d,e), a positive peak was measured (zone D) because of the formation of the contact area between the second water column. There was no signal in zone E for same reason as in zone A. Zones A, B and C provided information on the slug length and velocity of flow. The velocity of flow was easily estimated from the measured voltage difference at zones A and C. Peak to peak voltage from the self-powered sensor was ~0.201 V, and this value is almost similar to the value (~0.19 V) for the volumetric flow rate of 30 ml/min. Therefore, the velocity of the flow was 13.16 cm/s, which was converted from the volumetric flow rate to velocity in a channel with 2 mm × 1.9 mm cross sectional area. The length of the air slug was calculated by multiplying time by flow velocity. The times at zones B and C (0.503 s) provide the total length of the air slug. It was calculated as 6.61 cm at the cross sectional area of the channel with a total volume of ~0.25 ml which is acceptable data compared to 0.2616 ml. To verify, the velocity of the flow was measured from the frame numbers of the high speed movie which was recorded at 100 frames per 1 second. Twenty-six frames were captured, and this means that it took 0.26 s for the head of the air slug passing through the channel (Fig. 4b,c). From the time information, the velocity of the flow was measured as 13.8 cm/s and the calculated velocity of 13.16 cm/s from the self-powered sensor was reasonable. Although several factors were not considered in this experiment such as the friction between the water and the tubes and air gap compression at the channel, from these results, we successfully verified that FEG can be used as a self-powered slug detector in a small fluidic channel.

In summary, we have demonstrated electricity generation and air slug detection with the FEG in small channels using the two phase flow without any external sources. This novel fluidic active transducer has several strength points. First, it has very simple structure and fabrication method compared with previous works^6^,^7^,^8^,^9^,^10^. Second, until now, most of the electricity generators have been water vibrational generators^22^,^23^,^24^ but FEG can be operated under various channel flow conditions conforming to flows found in daily life and in nature. Furthermore, we expect that the FEG can be utilized not only as an energy harvester and self-powered sensor but also as analysis tools for fluidics such as fluid pattern estimation and various lab on a chip applications.

## Methods

### PDMS channel fabrication.

A mold for the channel was used by combining PMMA blocks and PTFE tubes (DAEKWANG HIFLON). Two types of long tubes, 1.8 cm and 3.6 cm lengths, with a 2.1 mm outside diameter were prepared for the channel. These tubes were inserted between PMMA blocks set a widths of 1.8 cm and 3.6 cm, respectively, and fixed with ketone tape. Then, unlinked 10:1 base and cross linker mixed PDMS (Sylgard-184, Dow Corning) solution was poured on the mold. This PDMS with mold was cured at 80 °C for 4 hours. After that, the PDMS and mold were detached, and inlet and outlet holes were drilled for tubing.

Materials and preparation of active transducer. In this experiment, the active transducer consisted of ITO glass, P4VP, and a silica gel layer. ITO glass was cut 1.8 cm by 4 cm, and 3.6 cm by 4 cm for the two types of channel lengths. P4VP solution 10 wt% was made with Poly (4-vinylphenol), Propylene glycol mono methyl ether acetate as the solvent and poly (melamine-co formaldehyde) methylated/ butylated as the cross linker (Sigma Aldrich) with a mass ratio 2:17:1. The silica gel film was formed by the sol-gel process. Silica sols were synthesized with H_2_O, Ethanol, tetraethyl silicate (TEOS) (Sigma Aldrich) and 1H, 1H, 2H, 2H- Perfluorooctyl-triethoxysilane (POTS) (Sigma Aldrich) in acid conditions. A 0.031 mass ratio of TEOS and POTS was used in this experiment. The ITO glass was spin coated with the 10 wt% P4VP solution at 2000 rpm for 30 s and annealed at 200 °C for 15 min. Then, the two samples of P4VP coated ITO glass were exposed to ultraviolet ozone (100 mW/cm^2^) for 30 min. Silica sol solution was spin coated with the same conditions as the P4VP. For the gelation of the silica sol layer, the substrate was baked at 200 °C. 99.4 degrees of contact angle were measured on the silica gel film (see Supplementary Fig. S1), and this hydrophobic property of this film assisted in the dewetting process when the water flowed in the fluidic channel.

Performance measurement equipment. The voltage and current were measured with an oscilloscope (DPO-2024) and a pico-ammeter (Keithley Model 6485), respectively. The water and air were controlled with a syringe pump (KdScientific Inc.).

## Additional Information

**How to cite this article**: Yang, Y.J. *et al.* Fluidic Active Transducer for Electricity Generation. *Sci. Rep.* **5**, 15695; doi: 10.1038/srep15695 (2015).

## Supplementary Material

Supplementary Information
