# Supplementary material for: Fluidic Active Transducer for Electricity Generation
Source: Sci Rep. 2015 Oct 29;5:15695. doi: 10.1038/srep15695 (PMC4625157; doi:10.1038/srep15695)
Supplement: Supplementary Information [file srep15695-s1.doc]

**Supplementary Information**

**Fluidic Active Transducer for Electricity Generation.**

YoungJun Yang^1^, Junwoo Park^1^, Soon-Hyung Kwon^1,3^ and Youn Sang Kim^1,2^*

[*] 1. Program in Nano Science and Technology,

Graduate School of Convergence Science and Technology, Seoul National University

1 Gwanak-ro, Gwanak-gu, Seoul 151-742, Republic of Korea. E-mail: [younskim@snu.ac.kr](mailto:younskim@snu.ac.kr)

2. Advanced Institutes of Convergence Technology,

864-1 Iui-dong, Yeongtong-gu, Suwon-si, Gyeonggi-do 443-270, Republic of Korea

3. Flexible Display Research Center, Korea Electronics Technology Institute,

25 Saenari-ro, Bundang-gu, Seongnam-si, Gyeonggi-do 464-816, Republic of Korea.


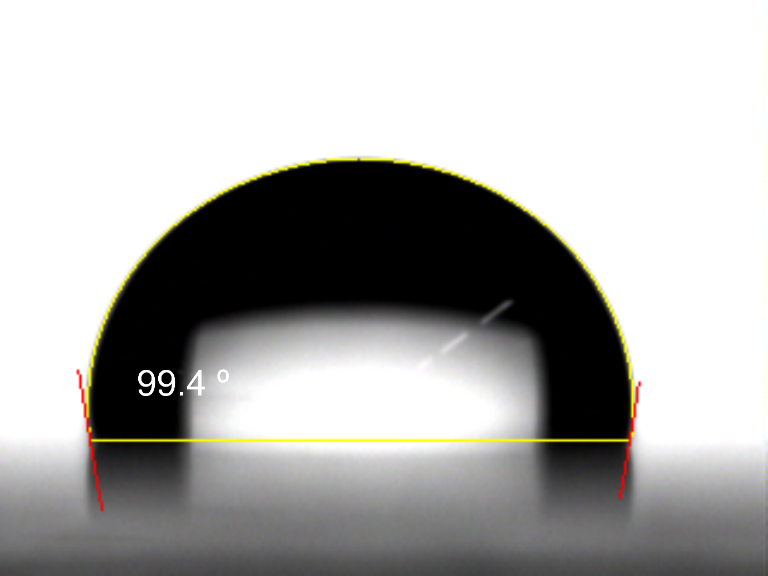


**Supplementary Figure S1 | Contact angle image**. A contact angle of 99.4 degrees was measured for a deionized water droplet on the silica gel film.


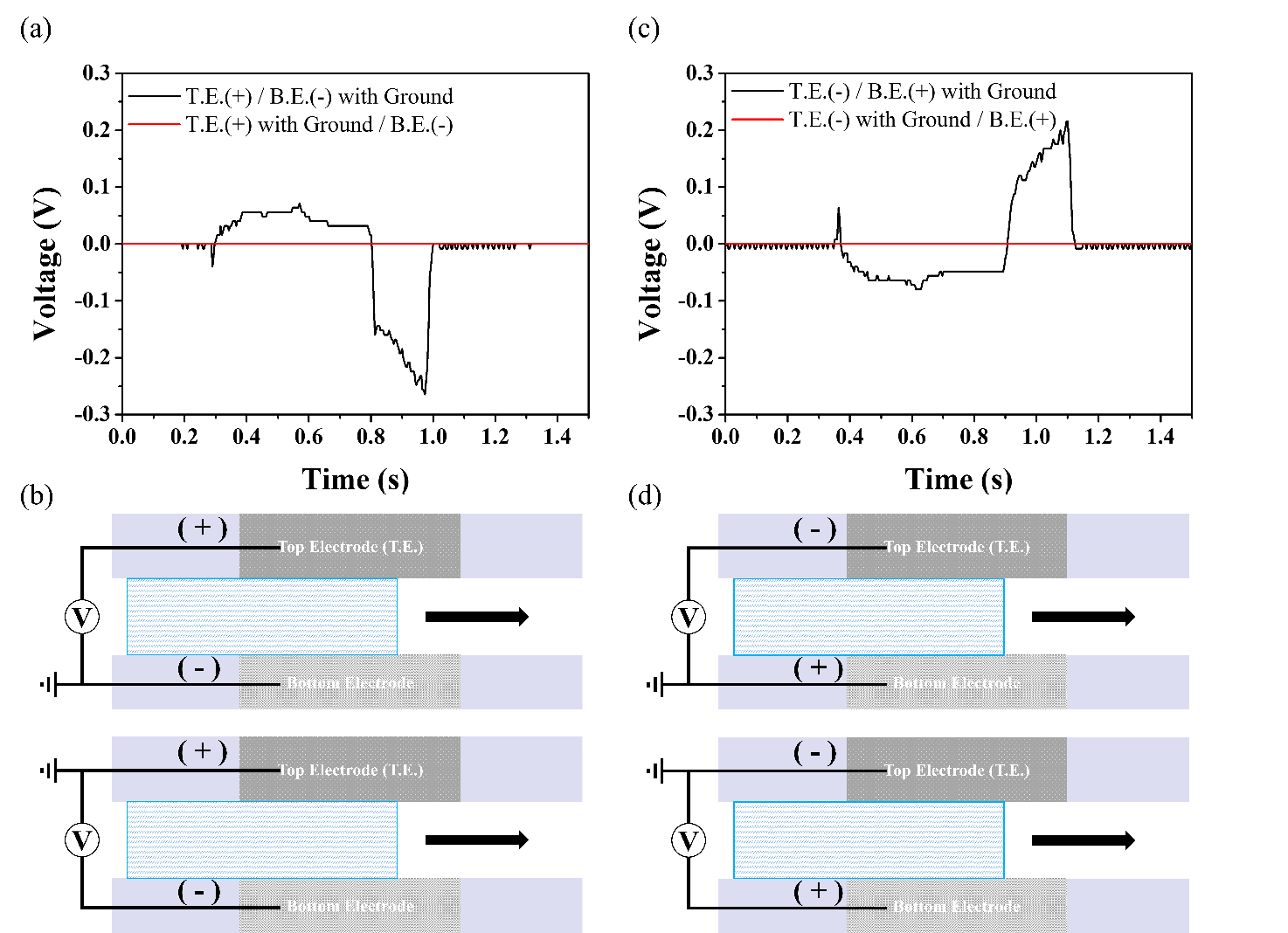


Supplementary Figure S2 | Potential difference between ground and electrodes. (a), (b) The top and bottom electrodes were connected with the (+) and (-) parts of the measurement, respectively. To check the major electrode, a ground was connected to the top and bottom electrodes. (c), (d) shows a reverse connection to verify the measured voltage data. In this experiment, the top electrode acts as a major factor in the FEG.


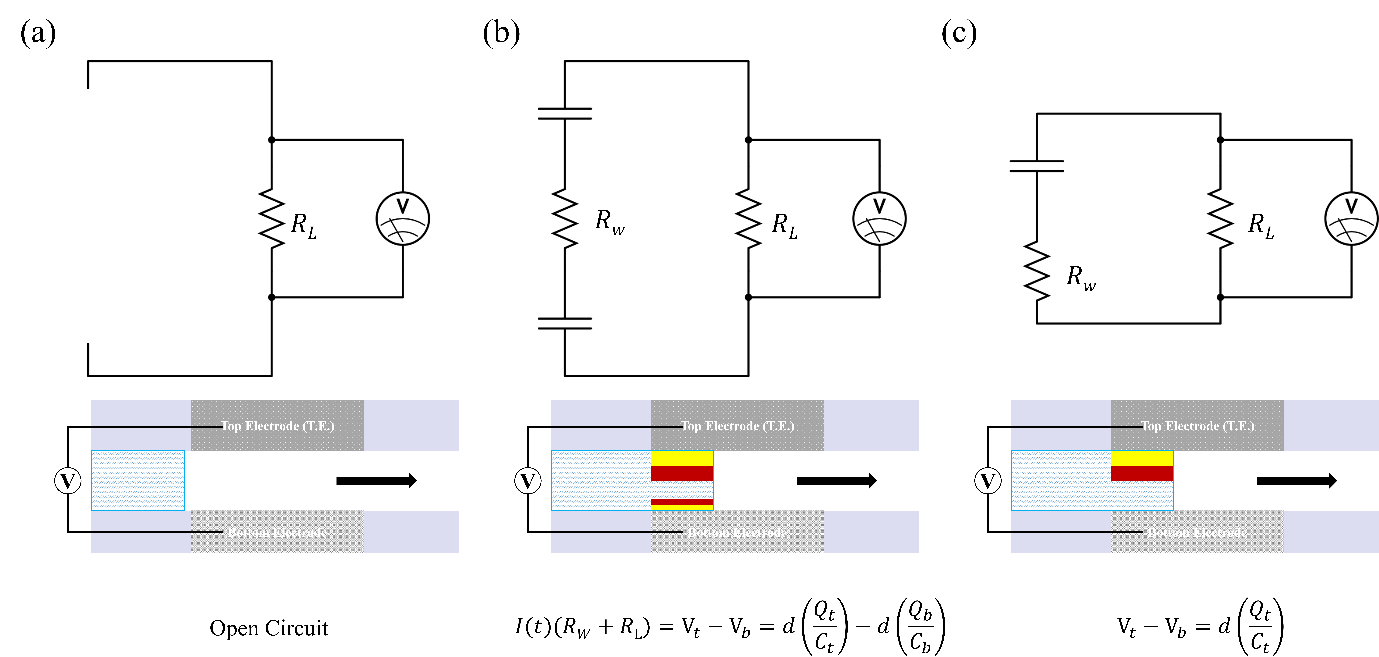


Supplementary Figure S3 | Circuit model simplification. The total system is an open circuit until the water column comes into contact with the top and bottom electrodes (a). With the contact of the water and electrodes, the circuit is shorted, and EDLs are formed at the top and bottom electrodes (b). At that time, voltage difference follows the equation^10^ shown in (b), where *R_W_* and *R_L_* are the resistance of the water and circuit like the voltmeter and wires; *V_t_* and *V_b_* are the voltages on *C_t_* and *C_b_.* From Supplementary Figure S2, the bottom electrode effect is small enough to be negligible. Thus, the voltage difference can be expressed as the equation shown in (c).


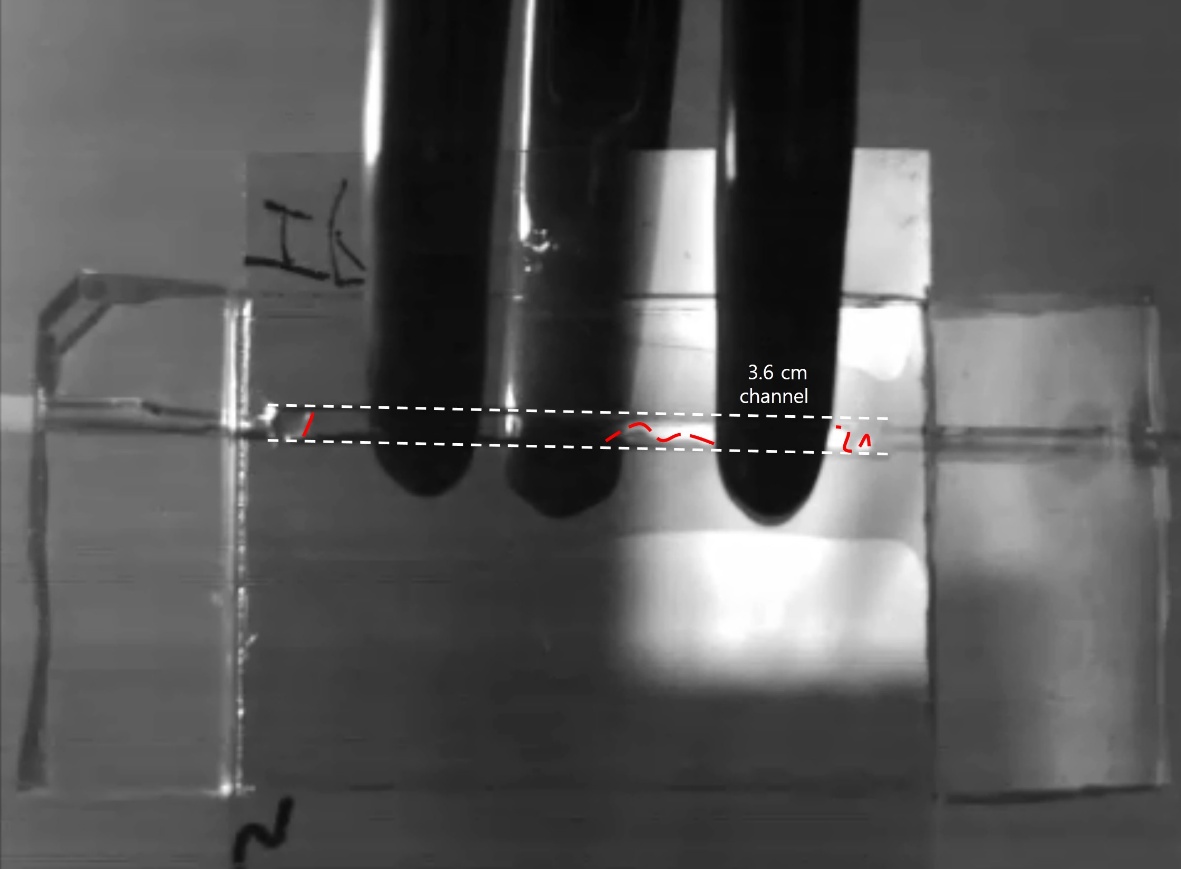


Supplementary Figure S4 | Captured image of the super-high speed camera movie. Remaining tiny water droplets on the electrode (Edge of the water droplets were marked as red dot line).

**Supplementary Note**

**Supplementary Note 1. Energy conversion efficiency of the FEG with two phase flow of water and air.**

The energy conversion efficiency of the FEG is given by ^1, 2^


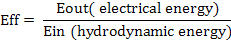

The 3.6 cm long FEG and the 10 cm long water columns generated ~1 nJ per a water column. Assuming that the 2 units of 3.6 cm long water columns and 1 unit of air slug pass through a transducer in a second, the 15 generators in 1 m long tube can produce about 30 nJ (E_out_). The E_in_ can be expressed as pressure by flow rate. In this experiment, about 40 mbar of pressure and 30 ml/min of flow rate were used. The calculated hydrodynamic energy was about 2 mJ. Therefore, the efficiency of the FEG is 1.5 %. However, when the FEG is more integrated, the E_out_ can be much enhanced.

1.Nguyen T, Xie Y, de Vreede LJ, van den Berg A, Eijkel JC. Highly enhanced energy conversion from the streaming current by polymer addition*. Lab chip* **13**, 3210-3216 (2013).

2.Olthuis W, Schippers B, Eijkel J, van den Berg A. Energy from streaming current and potential. *Sensor Actuat. B-Chem.* **111-112**, 385-389 (2005).
